# Simple fully convolutional network to detect Alzheimer’s Disease

**DOI:** 10.1002/alz.088097

**Published:** 2025-01-09

**Authors:** Ninad Aithal, Neelam Sinha

**Affiliations:** ^1^ Indian Institue of Sciences, Bengaluru, Karnataka India; ^2^ Centre for Brain Research (CBR), Indian Institute of Science, Bengaluru, Karnataka India

## Abstract

**Background:**

Deep Neural Networks (DNNs) have emerged as powerful tools in the biomedical field, particularly for the early detection of neurodegenerative diseases. Despite their complexity and data‐intensive nature, simpler fully connected Convolutional Neural Network (SFCN) architectures have shown effectiveness in accurately discerning between subjects affected by Alzheimer’s disease (AD) and healthy controls (HC). This model draws inspiration from the work of Peng H et al. and is trained on the openly available Alzheimer’s Disease Neuroimaging Initiative (ADNI) dataset.

**Method:**

In this study, RAW 3T T1‐weighted MPRAGE MRI images with a sagittal acquisition plane and 3D acquisition type were carefully selected, amounting to 807 MRI images categorized into AD and CN classes. These images underwent brain extraction and linear registration (12 degrees of freedom) to a standard 2mm MNI template using FSL, resulting in uniform‐shaped scans of dimensions 1 × 91 × 109 × 91. The model comprises 5 blocks, each incorporating 2 × 3D CNN blocks, 3D batch normalization, and ReLU activation functions. A single MaxPooling layer doubles the channels at each CNN block, followed by a 3D pointwise CNN block reducing channels from 1024 to 256 while introducing non‐linearity. The final block includes 3D adaptive average pooling with a kernel size of 1, dropout set to 0.5, and 2 × 3D point‐ wise CNN layers acting as fully connected layers. This 13‐layer deep network is trained using Weighted Cross Entropy as the loss function and the Adam optimizer with a learning rate initialized to 0.00001. Training involved 100 epochs with a batch size of 16 and a 70‐10‐20 train‐validation‐test split.

**Result:**

The voxel‐based DNN, trained on 278 AD and 288 CN MRI images, achieves a remarkable 95% accuracy on a test dataset of 161, exhibiting precision and recall values of 0.96 and 0.94, respectively.

**Conclusion:**

The promising 95% classification accuracy on the ADNI dataset, encompass‐ ing over 800 subjects, underscores the efficacy of the SFCN in distinguishing between AD and NC subjects based on T1‐weighted MRI scans. This ap‐ proach holds potential for robust early detection of Alzheimer’s disease, con‐ tributing to advancements in neuroimaging‐based diagnostic methodologies.

